
JOURNAL INFORMATION
==============================
NLM Title Abbreviation: ScientificWorldJournal
Journal ID: ScientificWorldJournal
NLM Journal ID: 101131163
Journal ID: TSWJ

The Scientific World Journal

ISSN: 2356-6140
EISSN: 1537-744X
Publisher: Wiley

ARTICLE INFORMATION
==============================
PMCID: PMC6084231
PMID: 30147472
DOI: 10.1100/tsw.2001.160
Article ID: 618415
Article version: 1
Subjects: Short Report

The Antiapoptotic Effect of BHV-1 in Sorbitol-induced Apoptosis in MDBK Cells

Marfè G. 1
De Martino L. 2
Di Stefano C. 1 *
Iovane G. 2
Department of Experimental Medicine and Pathology, Medicine College, University “La Sapienza”- Rome, Italy
Department of Pathology and Animal Health-Infectious Diseases, Medicine Veterinary College, University “Federico II”- Naples, Italy
*C. Di Stefano: carladi_stefano@hotmail.com
Publication date: 2001
Electronic publication date: 2001 Oct 26
Volume: 1
First page: 35
Last page: 35
Copyright: Copyright © 2001 G. Marfegrave et al.
Copyright year: 2001
License: This is an open access article distributed under the Creative Commons Attribution License, which permits unrestricted use, distribution, and reproduction in any medium, provided the original work is properly cited.
License URL: https://creativecommons.org/licenses/by/3.0/

==============================
Miami *Nature Biotechnology* Short Reports
*TheScientificWorld* (2001) 1(S3), 35SR
ISSN 1532-2246; DOI 10.1100/tsw.2001.160

### THE ANTIAPOPTOTIC EFFECT OF BHV-1 IN SORBITOL-INDUCED APOPTOSIS IN MDBK CELLS

Marfè, G.[1], De Martino, L.[2], Di Stefano, C.[1,*], and Iovane, G.[2]

[1]Department of Experimental Medicine and Pathology, Medicine College, University "La Sapienza"- Rome;
[2]Department of Pathology and Animal Health-Infectious Diseases, Medicine Veterinary College, University "Federico II"- Naples
* carladi_stefano@hotmail.com

**INTRODUCTION**. Apoptosis is a mechanism of eukariotic cell suicide which plays a major role in many physiological and pathological processes. Many viruses have developed different pathways to induce or inhibit the apoptotic process sufficiently to allow the completion of viral replication. In this report, we show that bovine herpes virus type-1(BVH-1) is able to inhibit the sorbitol-induced apoptosis in Madin-Darby bovine kidney (MDBK) cells.

Our results demonstrate that DNA fragmentation disappears in the cells after 7 hrs post-infection.

**METHOD.** It was evaluated the expression of p53, bcl-2 and bax genes at indicated times of post-infection. For RT-PCR studies, total RNAs were extracted from treated and untreated MDBK cells with Trizol (GIBCO BRL). The reaction products were visualized via electrophoresis using 20 µl of reaction mixture at 80 volts in a 1,5% agarose gel, containg 0.5 µg/ml ethidium bromide. The gels were then examined on UV light-box and photographed.

**RESULTS.** In MDBK cells, infected with BHV-1 and treated with sorbitol, the expression of p53 gene was reduced after 7 hrs post-infection until it disappeared after 11 hrs post-infection; while the expression of bcl2 and bax genes gradually was decreased up to 3 hrs until it disappeared after 5 hrs post-infection.

**DISCUSSION.** In the present study, we have investigated expression of representative genes encoding apoptosis.promoting and-inhibiting factors during the course of sorbitol-induced apoptosis in MDBK cells, infected with BHV-1. In order to understand the molecular mechanisms, we have investigated the role of p53 and bax as apoptosis-promoting factors and the role of bcl-2 as an apoptosis inhibiting-factor. p53 is involved in the onset of sorbitol-induced apoptosis in MDBK cells, infected with BHV-1, since the level of the expression of the gene was decreased after 7 hrs post-infection. Thus, sorbitol-induced apoptosis in MDBK cells, infected with BHV-1, appears to be p53-dependent.

At the same time, the expression of the bcl-2 and bax genes decreased after 3 hrs post-infection. This results indicate that a viral product can suppress the expression of bax, and bcl-2 can regulate the bax-induced caspase activation.

**REFERENCES.**

1. Steller, H. (1995) Science 267, 1445-1449
2. Gillet, G. et al. (1996) Trends Microbiol. 4, 312-317
3. Kitanaka, C., Namiki, T., Noguchi, K., Mochizuki, T., Kagaya, S., Chi, S., Hayashi, A., 4. Asai, A., Tsujimoto, Y., and Kuchino, Y.(1997) Oncogene 15, 1763-1772
